# Gas Plasma Technology Augments Ovalbumin Immunogenicity and OT‐II T Cell Activation Conferring Tumor Protection in Mice

**DOI:** 10.1002/advs.202003395

**Published:** 2021-03-08

**Authors:** Ramona Clemen, Eric Freund, Daniel Mrochen, Lea Miebach, Anke Schmidt, Bernhard H. Rauch, Jan‐Wilm Lackmann, Ulrike Martens, Kristian Wende, Michael Lalk, Mihaela Delcea, Barbara M. Bröker, Sander Bekeschus

**Affiliations:** ^1^ ZIK plasmatis Leibniz Institute for Plasma Science and Technology (INP) Felix‐Hausdorff‐Str. 2 Greifswald 17489 Germany; ^2^ Department of General Visceral Thoracic and Vascular Surgery University Medicine Greifswald Sauerbruchstr. DZ7 Greifswald 17475 Germany; ^3^ Department of Immunology University Medicine Greifswald Sauerbruchstr. DZ7 Greifswald 17475 Germany; ^4^ Institute of Pharmacology (C_Dat) University Medicine Greifswald Felix‐Hausdorff‐Str. 1 Greifswald 17489 Germany; ^5^ CECAD proteomics facility University of Cologne Joseph‐Stelzmann‐Str. 26 Cologne 50931 Germany; ^6^ ZIK HIKE University of Greifswald Fleischmannstr. 42–44 Greifswald 17489 Germany; ^7^ Institute of Biochemistry University of Greifswald Felix‐Hausdorff‐Str. 4 Greifswald 17489 Germany

**Keywords:** kINPen, ovalbumin, oxPTM, ROS, vaccines

## Abstract

Reactive oxygen species (ROS/RNS) are produced during inflammation and elicit protein modifications, but the immunological consequences are largely unknown. Gas plasma technology capable of generating an unmatched variety of ROS/RNS is deployed to mimic inflammation and study the significance of ROS/RNS modifications using the model protein chicken ovalbumin (Ova vs oxOva). Dynamic light scattering and circular dichroism spectroscopy reveal structural modifications in oxOva compared to Ova. T cells from Ova‐specific OT‐II but not from C57BL/6 or SKH‐1 wild type mice presents enhanced activation after Ova addition. OxOva exacerbates this activation when administered ex vivo or in vivo, along with an increased interferon‐gamma production, a known anti‐melanoma agent. OxOva vaccination of wild type mice followed by inoculation of syngeneic B16F10 Ova‐expressing melanoma cells shows enhanced T cell number and activation, decreased tumor burden, and elevated numbers of antigen‐presenting cells when compared to their Ova‐vaccinated counterparts. Analysis of oxOva using mass spectrometry identifies three hot spots regions rich in oxidative modifications that are associated with the increased T cell activation. Using Ova as a model protein, the findings suggest an immunomodulating role of multi‐ROS/RNS modifications that may spur novel research lines in inflammation research and for vaccination strategies in oncology.

## Graphical abstract

Successful vaccination requires increased immunogenicity of antigens. Cold physical plasmas produce reactive oxygen and nitrogen species capable of eliciting oxidative protein modifications. Oxidation of ovalbumin (Ova) shows enhanced T cell activation, elevated numbers of antigen‐presenting cells, and decreases tumor burden in vivo upon vaccination.

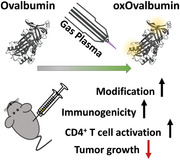

## 1 Introduction

Reactive oxygen and nitrogen species (ROS/RNS) play a multifaceted role in biology.^[^ ^1^ ^]^ They are part of the ancient immune defense mechanisms to protect from infection. At homeostatic levels, ROS/RNS also are versatile signaling molecules involved in antioxidant defense pathways, cell differentiation, and migration.^[^ ^2^ ^]^ At supra‐physiological levels during inflammation, however, ROS/RNS damage cells and tissues. Chronic inflammation and ROS/RNS generation is associated with several common diseases, such as autoimmunity, cardiovascular disease, and carcinogenesis.^[^ ^3^, ^4^ ^]^ While the unleashed activity of ROS‐generating cells and immunopathological mechanisms in inflammation‐associated diseases has been widely investigated in the past decades,^[^ ^5^, ^6^, ^7^ ^]^ the specific roles of ROS/RNS‐modified protein antigens are underexplored. Evidence for their significance is gained from reports on, for instance, oxidized low‐density lipoprotein as a putative biomarker in diabetes and cardiovascular disease.^[^ ^8^, ^9^ ^]^ A similar suggestion was made for oxidatively modified laminin in atherosclerosis.^[^ ^10^ ^]^ In these conditions, it is hypothesized that ROS/RNS‐mediated protein modifications may generate novel immunoepitopes or break tolerance against existing epitopes, which contributes to autoinflammation and autoimmunity.^[^ ^11^, ^12^ ^]^ While current therapeutic strategies aim to decrease ROS/RNS levels to avoid misguided immunity, the same is encouraged in some research lines in oncology. This seeming contradiction relates to the fact that tumors actively suppress immune responses targeted against tumor antigens, and ROS/RNS may revert this action as recently suggested in a vaccination study showing increased protection in ovarian cancer patients.^[^ ^13^ ^]^

The ROS/RNS chemistry and reaction pathways are complex as myeloid cells and partially also stromal cells release a plethora of different enzymatically generated ROS/RNS during inflammation. For instance, the nitric oxide synthase (NOS) produces nitric oxide (NO), while NADPH oxidases (NOX) generate superoxide (O_2_^−^), and both agents can combine to yield peroxynitrite (^−^ONOO). Superoxide can also spontaneously disproportionate to hydrogen peroxide (H_2_O_2_), a process amplified by the enzyme superoxide dismutase (SOD). The enzyme myeloperoxidase (MPO) is known to generate hypobromous acid, hypochlorous acid, and hypothiocyanite.^[^ ^14^ ^]^ Hypochlorite, in turn, contributes to the generation of atomic and singlet oxygen (O) and hydroxyl radicals (OH),^[^ ^15^ ^]^ while the latter is predominantly generated via the Fenton reaction of H_2_O_2_ with ferrous iron as catalyst. Some or even the complete selection of mentioned agents are present during inflammation. However, it is challenging to produce them simultaneously by chemical means to model the inflammatory environment.

Gas plasma, an electron‐impact and photon‐driven technology, bridges this gap. This partially ionized gas generates diverse ROS/RNS concurrently and in a spatially controlled manner.^[^ ^16^ ^]^ In physics, the term gas plasma generally includes also hot plasmas not suitable for biomedical applications. Hence, we here use the term gas plasma as a synonym for physical plasmas that operate at about body temperature and do not cause thermal harm to cells and tissues. Other synonyms of the term gas plasma used in applied plasma physics in biomedical fields are cold (physical) plasma, nonthermal plasma, (cold) atmospheric (pressure) plasma, tissue‐tolerable plasma, discharge plasma, and low‐temperature plasma. In gas plasma jets, a noble gas is excited by a high‐frequency electrode, and the excited noble gas species transfer their chemical energy to oxygen and nitrogen in the ambient air, generating reactive oxygen and nitrogen species, respectively. Recent leap innovations facilitated this technology to generate highly reactive gas plasmas at body temperature, allowing the study of ROS/RNS without thermal effects in the medical field.^[^ ^17^ ^]^ Not only due to its antibacterial properties,^[^ ^18^ ^]^ this technology is successfully applied for studying the promotion of ROS/RNS‐related wound healing in animal models ^[^ ^19^ ^]^ as well as in patients.^[^ ^20^ ^]^ As ROS/RNS have hormetic properties, being stimulating at low and toxic at high doses,^[^ ^21^ ^]^ the technology is increasingly investigated to treat cancer, especially of the skin.^[^ ^22^, ^23^, ^24^, ^25^, ^26^, ^27^ ^]^ We have recently provided evidence in mice that gas plasma treatment reduces skin cancer ^[^ ^26^ ^]^ and first patients suffering from actinic keratosis ^[^ ^28^ ^]^ and end‐stage head and neck cancer have benefited from gas plasma therapy.^[^ ^29^ ^]^ Strikingly, we and others identified gas plasma treatment to have an immunological dimension ^[^ ^30^, ^31^ ^]^ by inducing the immunogenic cancer cell death (ICD ^[^ ^32^ ^]^) in vitro ^[^ ^33^, ^34^ ^]^ and in vivo.^[^ ^35^, ^36^ ^]^ Several reports have moreover suggested using gas plasma technology as anticancer agent against internal tumors ^[^ ^37^ ^]^ originating from, for instance, breast,^[^ ^38^, ^39^, ^40^ ^]^ pancreatic,^[^ ^41^, ^42^, ^43^ ^]^ colon,^[^ ^44^, ^45^, ^46^ ^]^ liver,^[^ ^47^, ^48^, ^49^ ^]^ central nervous,^[^ ^50^, ^51^, ^52^ ^]^ ovarian,^[^ ^53^, ^54^, ^55^ ^]^ and prostate^[^ ^56^, ^57^, ^58^ ^]^ tissue. These and other studies also clearly demonstrated the importance of short‐lived ROS/RNS in the induction of ICD. Although unambiguously identifying every single type of ROS/RNS in the gas plasma is currently limited by the lack of technical tools, the composition of gas plasma‐derived ROS/RNS landscapes and subsequent biological responses can be controlled by modifying the gas composition fed into a gas plasma jet.^[^ ^59^, ^60^ ^]^ The different landscapes allow identifying sets of effector ROS/RNS linked to the effects observed.

We here aimed to understand the immunological consequences of a ROS/RNS‐modified model protein, chicken ovalbumin (Ova), using preformed anti‐Ova T cells from OT‐II mice. To generate a versatile array of inflammation‐related ROS/RNS, gas plasma technology was used for Ova oxidation (oxOva). We identified enhanced T cell activity towards oxOva ex vivo and in vivo and found a tumor‐protective action of oxOva when given as a vaccine to mice challenged with Ova‐expressing melanoma cells. Two different gas plasmas setups were used, one operated with argon (ox I) and another operating with helium/oxygen (ox II) gas. Each generated a distinct pattern of ROS/RNS as analyzed in the plasma gas phase, treated liquids, and modified proteins using mass spectrometry. By finding that ox II was superior to ox I in amplifying T cell responses and linking this to the physico‐chemical analysis of both gas plasma setups, singlet delta oxygen and atomic oxygen reaching the target are components suggested to be held responsible for promoting the immunogenicity of ovalbumin in our model systems.

## 2 Results

### 2.1 Gas Plasma‐Generated ROS/RNS Chemistry and Protein Modification

**Figure 1:**
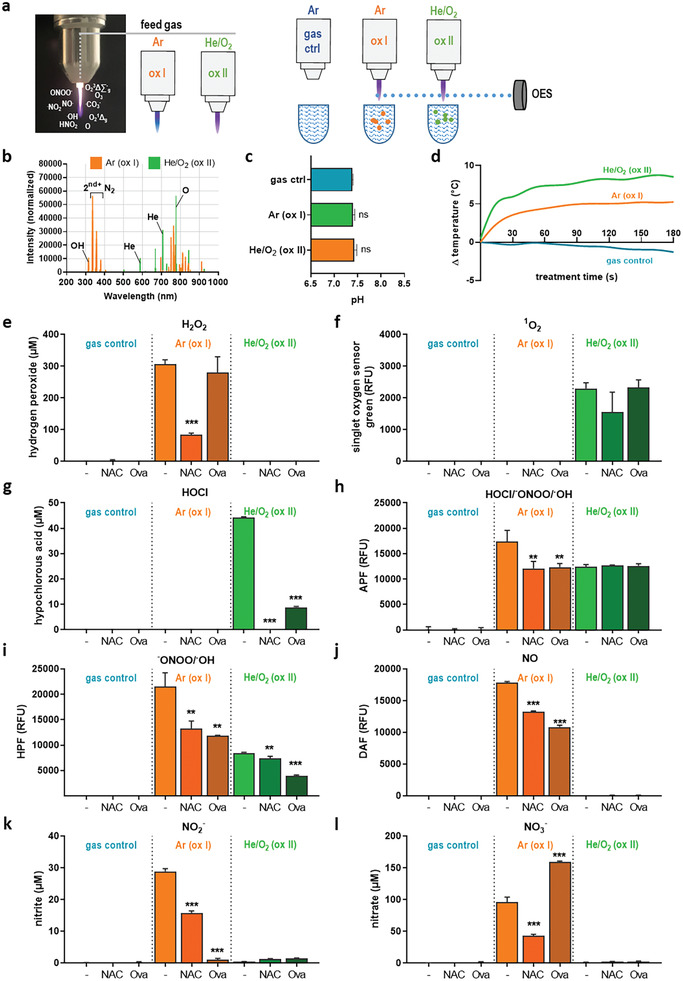
ROS/RNS fingerprint of two distinct gas plasma modes. a) Image of the atmospheric pressure plasma jet kINPen used in this study, and schematic of the two gas modes employed to generate different types of plasma; b) optical emission spectroscopy (OES) of the two plasma modes showing distinct peak characteristics representing different types of reactive atoms and molecules such as reactive nitrogen species (RNS), hydroxyl radicals (OH), and atomic oxygen (O); c) pH of PBS measured after gas plasma treatment; d) temperature kinetic of PBS during gas plasma exposure; e–l) major reactive species analyzed in control and gas plasma‐treated PBS in the presence or absence of ovalbumin (Ova) or *n*‐acetylcysteine (NAC), showing levels of e) hydrogen peroxide (H_2_O_2_), f) singlet oxygen (^1^O_2_), g) hypochlorous acid (HOCl), h) APF, i) HPF, j) DAF, k) nitrite (NO_2_^−^), and l) nitrate (NO_3_^−^), Data are representative of several experiments; statistical analysis was performed using one‐way anova (* *p* < 0.05; ** *p* < 0.01; *** *p* < 0.001); Ar = argon; He = helium; ctrl = control.

**Figure 2:**
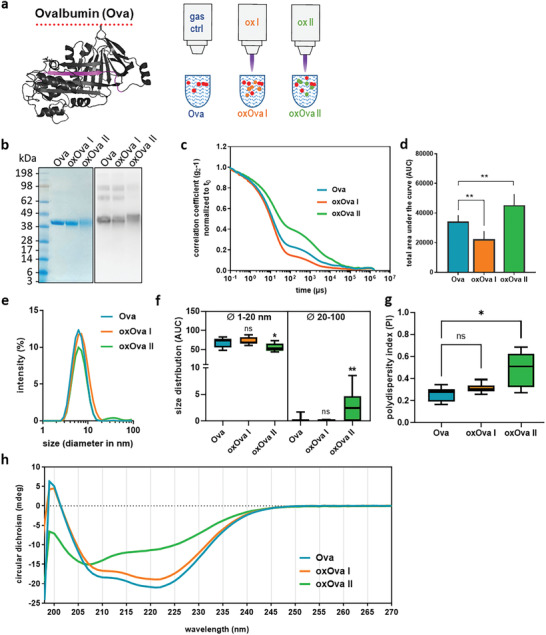
Gas plasmas‐derived modifications of Ova protein morphology. a) Crystal structure of Ova and schematic indicating the gas plasma treatment of Ova; b) representative coomassie‐stained gel and western blot of Ova and oxOva showing different staining patterns. c,d) photon correlation spectroscopy and correlation coefficients c) showing smaller and larger Ova structures for d) oxOva I and oxOva II (*n* = 4), respectively; e) intensity‐weighted hydrodynamic diameter and f) area under the curve (AUC) quantification of this parameter suggesting modest but significant aggregation with oxOva II (*n* = 3); g) polydispersity index (PDI) of correlogram showing more diverse particles with oxOva II (*n* = 3); h) structural properties and secondary folding measured using circular dichroism spectroscopy (*n* = 2). Statistical analysis was performed using one‐way anova (* *p* < 0.05; ** *p* < 0.01; *** *p* < 0.001); ns = not significant.

We aimed to investigate the immunological consequences of ROS/RNS‐derived protein modifications using chicken ovalbumin (Ova) as a model protein. To mimic a multi‐ROS/RNS environment, gas plasma, an electron‐impact and photon‐driven technology, was employed using an atmospheric pressure plasma jet. As feed gas, either argon (ox I) or helium/oxygen (ox II) was used (**Figure** **1**a, left panel), and the gas plasma, as well as the gas plasma‐treated liquid, was analyzed (Figure 1a, right panel). To distinguish distinct ROS/RNS fingerprints in these two modes, optical emission spectroscopy (OES) identified the ox I set up to be rich in the lines of the second positive system of nitrogen that are responsible for RNS formation as well as hydroxyl radical (OH). In contrast, the ox II setup showed enrichment of atomic oxygen (O) (Figure 1b). These two modes were subsequently used to treat a liquid (PBS) spiked with Ova or not. Alternatively, mock gas treatment alone (with the plasma being switched off) served as control. Only one mock condition was used as both were shown to not have an effect in pilot experiments. The gas plasma treatment did not affect the pH of a phosphate‐saline solution (Figure 1c), while modest changes in the temperature of this solution (baseline temperature: 15 °C) were observed (Figure 1d). Subsequently, the analysis of a selection of primary and secondary reactive species in the liquid was performed in the absence or presence of Ova or *n*‐acetylcysteine (NAC). For hydrogen peroxide (H_2_O_2_), only ox I but not ox II conditions yielded this secondary oxidant mostly derived from hydroxyl radicals, and the presence of NAC but not Ova reduced significant amounts of H_2_O_2_ (Figure 1e). For singlet delta oxygen (^1^O_2_), a marked elevation in the ox II but not the ox I condition was revealed with negligible scavenging ability of Ova (Figure 1f). By contrast, both Ova and NAC scavenged significant amounts of hypochlorous acid (HOCl) generated in the ox II but not ox I mode (Figure 1g). Aminophenyl fluorescein (APF) and hydroxyphenyl fluorescein (HPF) sense peroxynitrite (^−^ONOO) and hydroxyl radicals (^.^OH), while APF also senses HOCl.^[^ ^61^ ^]^ Both ox I and ox II increased fluorescence of APF, while NAC and OVA significantly reduced signal intensities only in ox I (Figure 1h). Ova and NAC reduced HPF fluorescence following ox I and ox II treatment (Figure 1i). Therefore, the data of both APF and HPF suggested the generation of hydroxyl radicals and peroxynitrite, which was confirmed for hydroxyl radicals (Figure S1a, Supporting Information) and is in line with previous research on this jet's chemistry.^[^ ^62^, ^63^ ^]^ Increased fluorescence of diaminofluoresceins (DAF) indicates nitric oxide (NO).^[^ ^64^ ^]^ Under the ox I but not ox II condition generating emission lines of the second positive nitrogen system capable of eliciting RNS (Figure 1b), ox I but not ox II treatment generated NO (Figure 1j) and the NO‐related products nitrite (Figure 1k) and nitrate (Figure 1l). Interestingly, the presence of Ova during the treatment modulated the levels of nitrite (NO_2_^−^) and nitrate (NO_3_^−^) levels in both ox I and ox II conditions. Moreover, NAC significantly scavenged NO, NO_2_^−^, and NO_3_^−^, while the presence of Ova led to a significant increase of the latter. These data pointed to two distinct ROS/RNS chemistries of the ox I and ox II condition and the Ova protein interfering with the decay kinetics of the reactive species deposited by the plasma jet. Subsequently, Ova treated with either the ox I (oxOva I) or ox II (oxOva II) gas plasma setting was analyzed (**Figure** **2**a). Although treatment with ox I and ox II seemingly decreased the presence of native Ova in nonreducing SDS‐PAGE (Figure 2b, left panel), Western Blot analysis confirmed the presence of the protein (Figure 2b, right panel). To analyze possible protein degradation or aggregation, dynamic light scattering was performed. The correlogram of oxOva II showed a shorter light scatter decay time, and the one of oxOva I was longer (Figure 2c), both being significantly different from native Ova (Figure 2d). In the presence of NAC, correlograms of Ova, oxOva I, and oxOva II were similar (Figure S1b, Supporting Information), suggesting a ROS/RNS dependent mechanism of the changes observed without NAC. Moreover, these findings indicated changes in the reflective properties of the protein solution following gas plasma treatment. Diameter measurements confirmed these findings, and especially oxOva II showed a small but distinguishable second peak (Figure 2e), which area under the curve (AUC) was significantly larger compared to that of native Ova (Figure 2f). Collectively, this pointed to an overall subtle increase in protein aggregation in the ox II condition. The notion of a more diverse protein population in ox II was supported by calculating the polydispersity index (PDI) from the correlograms (Figure 2g). To characterize the gas plasma‐derived ROS/RNS and subsequently induced changes to Ova in terms of protein structure and folding, circular dichroism (CD) spectroscopy was performed. In CD spectroscopy measurement, *α*‐helical structures are indicated by a minimum signal at 209 nm and a shoulder at 222 nm.^[^ ^65^ ^]^ Characteristic for *β*‐sheets are peaks at 195 nm and a negative minimum at 217 nm.^[^ ^66^ ^]^ Gas plasma treatment of Ova led to a shift in the signal for minima and maxima as well as ellipticity (Figure 2h). This was pronounced in oxOva II, in which an increase of *α*‐helical structures and a reduction in *β*‐sheets were observed. Again, the presence of NAC abrogated these changes and showed similar CD spectra (Figure S1c, Supporting Information). In summary, we found both gas plasma modes to generate a distinct set of reactive species that subsequently affected the monomeric form and secondary structure of Ova, especially in the ox II condition being rich in atomic and singlet oxygen production.

### 2.2 Oxidized Ova Changes Antigen Uptake but not Activation in APCs Being Relevant for Mounting Activation of OT‐II T Cells

**Figure 3:**
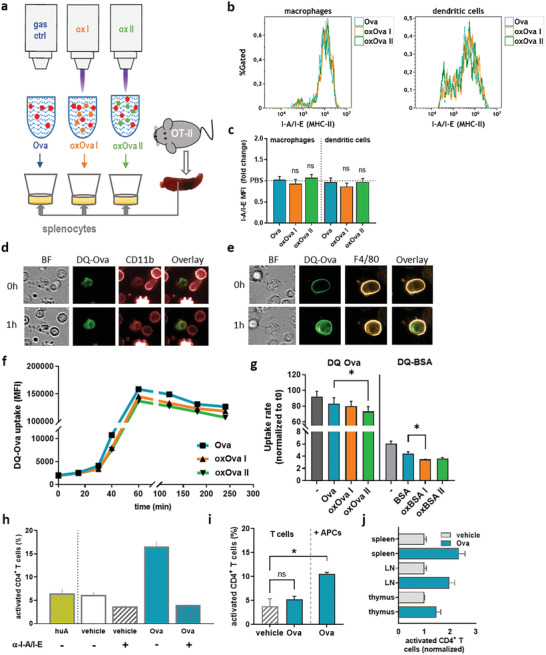
Oxidized Ova changes antigen uptake but not activation in APCs being relevant for mounting activation of OT‐II T cells. a) Setup of the gas plasma treatment of Ova and incubation with OT‐II derived splenocytes; b,c) representative flow cytometry intensity histograms of I‐A/I‐E (MHC‐II) expression on live macrophages and dendritic cells from splenocytes pulsed with either b) Ova, oxOVA I, or oxOVA II, and c) quantification thereof; d,e) visualization of DQ‐Ova (green) uptake and degradation in d) CD11b+ myeloid cells and e) F4/80^+^ macrophages; f,g) representative kinetic of the mean fluorescence intensity of DQ‐Ova fluorescing only upon uptake in I‐A/I‐E (MHC II)‐positive cells in presence of Ova, oxOva I, or oxOva II as determined using multicolor flow cytometry, and quantification thereof of DQ‐Ova (+Ova) or DQ‐BSA (+BSA) at g) 120 min; h) preincubation of splenocytes anti‐I‐A/I‐E antibodies abolished Ova‐induced activation of CD4^+^ T cells activation that was specific to chicken Ova but not to human albumin; i) activation of magnetically sorted CD4^+^ T cells in the presence of Ova or Ova+APCs; j) CD4^+^ T cell activation from different tissues after pulsing with Ova. Data representative of three independent experiments; statistical analysis was performed using one‐way anova (* *p* < 0.05); ns = not significant; scale bar = 20 µm.

ROS/RNS are agents plentifully released during inflammation, but the immunological consequences of ROS/RNS‐induced modifications are underexplored. To this end, we used gas plasma technology to oxidatively modify Ova (oxOva), and to incubate Ova‐specific T cells of splenocytes with Ova or oxOva before assessing their activity (**Figure** **3**a). Genetically engineered OT‐II mice harbor this specific set of CD4^+^ T helper cells that encode anti‐Ova T cell receptors. Splenocytes of OT‐II mice are a suitable model system to study the immunogenicity of Ova modifications. The hypothesis was that oxOva modulates the uptake, processing, and/or presentation of antigen by professional antigen‐presenting cells (APCs) such as macrophages and dendritic cells (DCs) via major histocompatibility complex (MHC) II to CD4^+^ T cells. Initially, the activation of viable macrophages (CD11b^+^/CD11c^+^/CD64^+^) and DCs (CD11b^+^/CD11c^+^/CD24^+^) being incubated with either Ova or oxOva was measured using flow cytometry analysis of the murine MHC II molecule I‐A/I‐E (Figure 3b). No significant I‐A/I‐E expression changes were found (Figure 3c), discouraging the idea of oxOva acting as an immediate danger‐signaling molecule in these cells. This was supported by western blot data bone marrow‐derived dendritic cells from wildtype C57BL/6 mice incubated with either Ova or oxOva II, and harvested 15, 30, and 60 min later for analyzing protein phosphorylation within MAPK‐related signaling pathways (Figure S1f, Supporting Information). None of the targets (Akt, Erk1/2, MSK2/3, RSK1/2) showed a significant change in oxOva II when compared against native Ova (Figure S1g, Supporting Information). To understand the specificity of T cell activation and the role of professional antigen‐presenting cells (APCs) in augmenting T cell activation, fluorescently labeled APC subtypes were initially investigated. Bodipy‐conjugated Ova (DQ‐Ova), an Ova‐aggregate, shows only background fluorescence since the fluorescence moieties quench each other when being in close vicinity. Upon uptake, however, DQ‐Ova is degraded, increasing its fluorescence intensity.^[^ ^67^ ^]^ We confirmed the uptake and degradation of this protein into CD11b^+^ myeloid cells in general (Figure 3d) and F4/80^+^ macrophages, specifically (Figure 3e). We then asked whether the uptake kinetics of DQ‐Ova in murine professional antigen‐presenting cells changed in the presence of Ova compared to oxOva I or oxOva II (Figure 3f). Interestingly, the uptake or processing of DQ‐Ova significantly declined in the presence of ox Ova II, suggesting competing uptake kinetics or preferential uptake via a specific route in APCs (Figure 3g). We were also able to recapitulate this finding in a second model protein, bovine serum albumin (BSA) compared to oxBSA I or oxBSA II in the presence of DQ‐BSA, and observed a less pronounced but still significant effect in ox I conditions (Figure 3g). To next ascertain the specificity of OT‐II CD4^+^ T cells to Ova‐derived peptides presented by APCs, the interaction between T cells and APCs was prohibited by using MHC‐II (I‐A/I‐E) blocking antibodies (Figure 3h). OT‐II T cell activation (% of CD69^+^/CD25^+^ cells) in splenocytes was observed only against chicken Ova but not human albumin (huA), and only in the absence of blocking antibody. Vice versa, magnetically isolated CD4^+^ T cells were not activated by Ova in the absence of APCs (Figure 3i). Investigating several lymphatic organs, T cell activation to Ova was maximal in splenocytes (Figure 3j). These data emphasized the suitability of the model and the specificity of OT‐II derived splenocytes for studying the effect of Ova modifications on CD4^+^ T cell activation.

### 2.3 Oxidation of Ova Augments Activation of OT‐II T Cells

**Figure 4:**
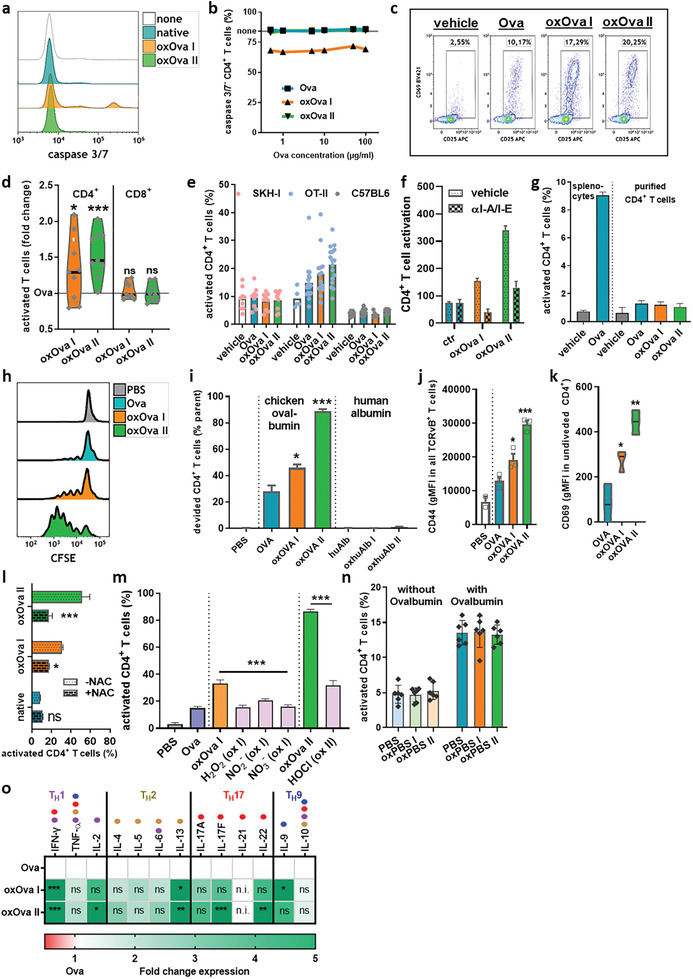
Oxidation of Ova augments the activation of OT‐II T cells. a) Representative overlay histograms and b) percentage of caspase 3/7^−^ cells 24 h after incubation (with either Ova, oxOva I, or oxOva II across a range of Ova concentrations; c) representative flow cytometry dot plots of OT‐II splenocytes‐derived CD4^+^ T cells and their activation (CD69^+^/CD25^+^) after stimulation with either Ova, oxOva I, or oxOva II at 24 h; d) quantification of activation of T cells from OT‐II (CD4^+^) or OT‐I (CD8^+^) splenocytes in response to either Ova, oxOva I, or oxOva II at 24 h, and shown as fold‐change of oxOva I and oxOva II to Ova; e) T cell activation of Ova‐specific CD4^+^ T cells from OT‐II mice‐derived splenocytes, wild type C57BL/6 mice‐derived splenocytes, or SKH‐1 mice‐derived splenocytes in response to either Ova, oxOva I, or oxOva II at 24 h; f) OT‐II splenocytes were cultured incubated with vehicle or I‐A/I‐E blocking antibodies prior to addition of Ova, oxOva I, and oxOva II, showing that CD4^+^ T cell activation was dependent on binding I‐A/I‐E on APCs; g) magnetically isolated CD4^+^ T cells alone (in absence of APCs) fail to show increased activation in response to oxOva I or oxOva II compared to CD4^+^ T cells within splenocytes, confirming the need of APCs to be present for the enhanced immunogenicity of oxOva in terms of T cell activation; h) 3d incubation of CFSE‐labeled OT‐II splenocytes with either h) Ova, oxOvaI, or oxOva II, and representative CFSE overlay, i) quantification of proliferating (CFSE^low^) cells including appropriate human albumin (huAlb) control, j) the intensity of the memory T cell marker CD44 in all TCRvB^+^ T cells, and k) staining intensity of the T cell activation marker CD69 in the nonproliferating (CFSE^hi^) T cell population; l) addition of NAC prior to gas plasma treatment of Ova abrogated the ability of oxOva I and oxOva II to augment CD4^+^ T cell activation in OT‐II splenocytes ex vivo; m) experimentally added (chemical) ROS/RNS such as hydrogen peroxide (H_2_O_2_, only generated in ox I), hypochlorous acid (HOCl, only generated in ox II), nitrite (NO_2_^−^, only generated in ox I), and nitrate (NO_3_^−^, only generated in ox I) supplied at the concentration matched to what gas plasma treatment generated in PBS alone failed to promote T cell activation to a similar extent compared to gas plasma‐derived mixtures of short‐lived ROS/RNS; n) PBS was left untreated or exposed to ox I (oxPBS I) or ox II (oxPBS II) gas plasma, added to OT‐II splenocytes, followed by addition of vehicle (‐ovalbumin) or Ova (+ovalbumin) 1 h later, and CD4^+^ T cell activation was measured 24 h later, showing the prerequisite of short‐lived ROS/RNS from the direct gas plasma treatment of Ova oxidation for augmented CD4^+^ T cell activation in OT‐II splenocytes ex vivo; o) T_H_ cell cytokine profile of Ova/oxOva I/oxOva II‐pulsed OT‐II‐splenocytes at 24 h with dominating cytokine signatures (T_H_1/T_H_2/T_H_17/T_H_9 segmentation) and annotated possible cytokine production (color code and dots). Data are representative of three independent experiments; statistical analysis was performed using one‐way or two‐way anova (* *p* < 0.05; ** *p* < 0.01; *** *p* < 0.001).

Several concentrations of Ova, oxOva I, and oxOva II were tested for their inherent toxicity to CD4^+^ T cells in terms of caspase 3/7 activation indicative of apoptosis (**Figure** **4**a). The quantification revealed a consistent but mild reduction of viability for oxOva I but not oxOva II (Figure 4b). Subsequently, the percentage of CD69^+^/CD25^+^ activated CD4^+^ T cells among all CD4^+^ T cells was analyzed ex vivo in splenocytes derived from OT‐II mice (Figure 4c). A significant fold‐change increase of T cell activation was observed for both ox Ova I and oxOva II when compared to Ova, which was specific for CD4^+^ OT‐II cells but not found in CD8^+^ T cells of OT‐I mice (Figure 4d). The effect of oxOva was identified only in T cells from OT‐II mice (harboring Ova‐specific CD4^+^ T cells) but not C57BL/6 or SKH‐1 wild type mice (Figure 4e). This suggested the oxPTM‐enhanced T cell activation ex vivo to take place preferentially in antigen‐specific cells. This striking finding suggested a role of oxidative protein modifications in T cell cross‐talk with APCs, as it was not observed in the presence of I‐A/I‐E (MHC II) blocking antibodies interfering with T cell‐APC interaction (Figure 4f) and in magnetically isolated T cells alone (absence of APCs) incubated with oxOva (Figure 4g). To underline the dominating role of oxidative protein modifications, we exposed Ova to pulsed electric fields, a physical property of gas plasma jets.^[^ ^68^ ^]^ The treatment could not increase the activation of CD4^+^ T cells among splenocytes above the level of native Ova alone (Figure S1d, Supporting Information), suggesting this physical parameter to not play a role in the effects observed. To further confirm T cell activation, proliferation studies were performed by labeling splenocytes with the cell‐tracer CFSE and analyzing the fluorescence distribution three days later (Figure 4h). The quantification of proliferated cells revealed a marked increase for oxOva I and oxOva II when compared to Ova (Figure 4i, left panel). Importantly, CD4^+^ OT‐II cells failed to proliferate not only in PBS (vehicle) controls but also in response to human albumin (huAlb) or gas plasma‐modified huAlb (oxhuAlb I and oxhuAlb II) (Figure 4j, right panel). Analysis of the expression of CD44, a marker of memory T cells, in all T cells exposed to either Ova, oxOva I, or oxOva II, the latter two showed significantly amplified CD44 intensities as determined at day 3 by flow cytometry (Figure 4j). Of note, quantifying the intensity of CD69, a T cell activation marker, in the nonproliferating portion of CD4^+^ OT‐II cells three days after challenge with either Ova, oxOva I, or oxOva II, a significant increase was observed (Figure 4k). This suggested that oxidative modifications of Ova not only spurred the proliferation of T cells but also led to the activation of T cells that did not proliferate. To provide evidence of the dominant role of ROS/RNS in these findings, the addition of the antioxidant NAC in plasma treated protein solution significantly decreased oxOva I and oxOva II‐induced T cell activation in OT‐II splenocytes (Figure 4l). To additionally ascertain that direct gas plasma treatment of Ova and its subsequent modifications via short‐lived ROS/RNS and not the mere presence of long‐lived ROS/RNS was a requirement for augmented T cell activation among splenocytes of OT‐II mice, two experiments were set up. In the first experiment, splenocytes were pulsed with Ova, oxOva I, oxOva II, or Ova treated with those concentrations of hydrogen peroxide (H_2_O_2_, only generated in ox I), nitrite (NO_2_^−^, only generated in ox I), nitrate (NO_3_^−^, only generated in ox I), and hypochlorous acid (HOCl, only generated in ox II) that exactly matched the concentrations yielded by gas plasma treatment in PBS alone, to disguise the effects of individual, long‐lived components. None of the agents recapitulated the increase in T cell activation as observed with ox I or ox II, except for a modest increased for HOCl (Figure 4m). In the second experiment, PBS without Ova was exposed to gas plasma or left untreated (PBS, oxPBS I, and oxPBS II). Incubation of splenocytes with oxPBS I or oxPBS II alone (in the absence of Ova) did not yield elevated T cell activation (Figure 4n, left panel). This underlined that the ROS/RNS alone did not promote T cell activation and only the Ova antigen or its oxidized counterpart oxOva did so. This important notion was underlined by gas plasma‐treating PBS first and adding Ova second before the Ova‐PBS or Ova‐oxPBS was added to splenocytes. No increase in T cell activation was observed (Figure 4n, right panel). These results strongly suggested that the direct gas plasma oxidation of Ova via short‐lived ROS/RNS, and not individual and well known long‐lived ROS/RNS alone, were required to achieve the strong stimulatory effects observed in OT‐II Ova‐specific T cells. Additionally, the finding of increased CD69 activation in nonproliferating T cells following exposure to oxOva (Figure 4k) indicated differential responses of CD4^+^ T cell subpopulations to oxOva‐derived peptides presented by APC, possibly being reflected in changes in the cytokine profile. To this end, we performed multiplex arrays to map the cytokine profiles that were accompanied by Ova or oxOva stimulation. In supernatants of splenocytes incubated with Ova or oxOva ex vivo for 24 h, a significant increase in interferon‐gamma (IFN*γ*) and interleukin (IL) 13 was identified (Figure 4o). T_H_17 cytokines, such as IL‐17F and IL‐22, were elevated as well. In general, it was the notion that oxOva II exerted more potent effects on cytokine release compared to oxOva I.

### 2.4 T Cell Response to Vaccination of OT‐II Mice with Ova or Oxidized Ova

**Figure 5:**
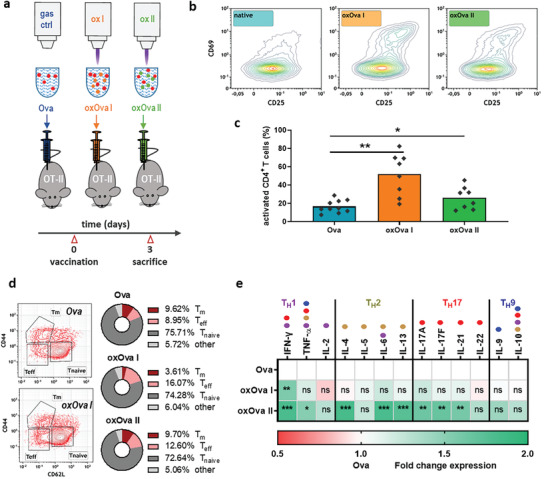
T cell response to vaccination of OT‐II mice with Ova or oxidized Ova. a) Setup of the gas plasma treatment of Ova and repeated injection of either Ova, oxOva I, or oxOva II into OT‐II mice; b) Representative CD69/CD25 expression and c) quantification of activated T cells from draining lymph nodes at d3; d) CD4^+^ T cell subpopulation analysis via CD44 and CD62L within the activated CD4^+^ parent population; e) T_H_ cell cytokine profile of cells of the draining lymph nodes of vaccinated OT‐II animals at 3d with dominating cytokine signatures (T_H_1/T_H_2/T_H_17/T_H_9 segmentation) and annotated possible cytokine production (color code and dots). Data are representative of at least 4 mice per group; statistical analysis was performed using one‐way or two‐way anova (* *p* < 0.05; ** *p* < 0.01; *** *p* < 0.001).

Up to this point, we used two different ROS/RNS environments generated by gas plasma to differentially oxidize Ova (oxOva I and oxOva II), and tested the consequences of incubation with either Ova or oxOva I or II in OT‐II derived splenocytes and T cell activation and proliferation ex vivo. To next analyze the ability of oxOva to exacerbate T cell activation in vivo (**Figure** **5**a), we rechallenged OT‐II mice with either Ova, oxOva I, or oxOva II, and three days later analyzed CD4^+^ T cell activity in the draining lymph nodes (Figure 5b). Both oxOva II and especially oxOva I vaccination but not oxOva + NAC (Figure S1e, Supporting Information) yielded significantly elevated numbers of activated T cells when compared to native Ova (Figure 5c), pointing to increased immunogenicity of the gas plasma‐treated protein ovalbumin. Among all activated CD4^+^ T cells, effector (oxOva II) and memory T cells (oxOva I) were found to a greater extent when compared to native activated T cells of animals vaccinated with Ova (Figure 5d). Similar to the results seen ex vivo, this pointed to a differential role of the T cell subpopulations, leading us to investigate the T_H_ cytokine profile in the minced draining lymph nodes of vaccinated animals in more detail (Figure 5e). For oxOva II, there was an increase in IL‐2 and IL‐17F as well as IL‐22. These results were strikingly similar to those obtained in the ex vivo experiments (Figure 4o). For all other targets investigated, oxOva I administered in vivo did not give any changes, while oxOva II additionally spurred the release of tumor necrosis factor (TNF) *α* and several other T_H_2 and T_H_17‐related cytokines. However, the most apparent congruency was found for IFN*γ* secretion being sharply elevated in both lymph nodes from oxOva I / ox Ova II vaccinated mice splenocytes incubated ex vivo with oxOva I / ox Ova II.

### 2.5 OxOva Vaccination of C57BL/6 Wild Type Reduced Melanoma Growth In Vivo

**Figure 6:**
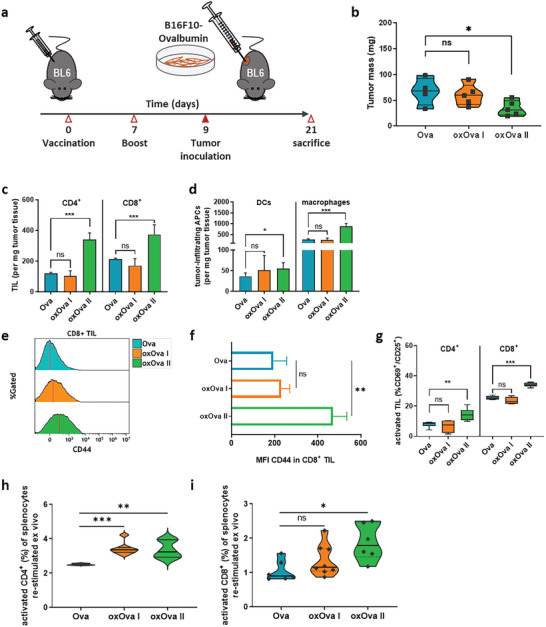
oxOva vaccination confers superior protection again B16F10‐Ova melanoma growth in vivo. a) Wild type C57BL/6 were vaccinated twice i.p. with 10 µg of either Ova, oxOva I, or oxOva II followed by subcutaneous inoculation of Ova‐expressing B16F10; b) tumor weights of individual animals in each group; c) tumor‐infiltrating lymphocytes (TIL) were elevated for CD4^+^ and CD8^+^ cells in the oxOva II vaccination regimen; d) number of tumor‐infiltrating APCs showed an increase of dendritic cells (DCs) and macrophages in the oxOva II vaccination regimen; e) representative flow cytometry intensity histograms of CD44 in CD8^+^ TIL and f) quantification; g) activation of CD4^+^ and CD8^+^ TIL was enhanced in the oxOva II vaccination regimen; h,i) splenocytes of tumor‐bearing animals receiving the respective Ova, oxOva I, or oxOva II vaccination were isolated and restimulated ex vivo with Ova and h) CD4^+^ and i) CD8^+^ T cell activation was analyzed 24 h later. Data are representative of three independent experiments and 6–8 mice per group; statistical analysis was performed using one‐way anova (* *p* < 0.05; ** *p* < 0.01; *** *p* < 0.001).

Vaccination of OT‐II mice with oxOva generated a marked increase in IFN*γ* release, a molecule known for its antitumor effects. To test the functional consequences of gas plasma‐oxidized Ova in wild type mice with no pre‐existing anti‐Ova adaptive immunity, C57BL/6 mice were vaccinated two times with either Ova or oxOva, followed by subcutaneous inoculation of Ova‐expressing B16F10 syngeneic melanoma cells (**Figure** **6**a). OxOva II but not oxOva I immunization led to a significantly impaired tumor growth (Figure 6b), pointing to an enhanced adaptive antitumor immune response mediated by the oxidized compared to the native form of Ova. The analysis of tumor‐infiltrating T cells showed increased numbers of CD4^+^ and CD8^+^ T cells in the tumor microenvironment (Figure 6c). OxOva vaccination also was accompanied by significantly elevated numbers of intratumoral dendritic cells and macrophages (Figure 6d). Additionally, in CD8^+^ cytotoxic T cells, the expression of the memory T cell marker CD44 (Figure 6e) was found to be increased in tumors of mice that had received oxOva II vaccination (Figure 6f). This corroborated our analysis of the T cell activation profile, which was found to be enhanced in both CD4^+^ and CD8^+^ T cells but only for oxOva II and not oxOva I vaccination (Figure 6g). These findings suggested that oxOva II led to a more pronounced generation of Ova‐specific T cells that, in turn, contributed to decelerated growth of Ova‐expressing melanoma cells in wild type mice in vivo. Finally, to confirm the improved generation and activation of novel anti‐Ova T cell entities, splenocytes of tumor‐bearing wild type mice receiving oxOva vaccination were restimulated with Ova ex vivo. The analysis of the percentage of activated T cells 24 h later showed a significantly increased activation in the CD4^+^ helper cell subpopulation for both oxOva I and oxOva II vaccinated mice (Figure 6h). In contrast, a more pronounced activation within the CD8^+^ cytotoxic subpopulation was observed for oxOva II only (Figure 6i). Interestingly, a modest but significant enhancement of T cell activation was also observed upon restimulation with the melanoma antigen MART‐1 (data not shown), a finding that warrants further investigation in future studies. Collectively, these results suggest that gas plasma‐derived ROS/RNS increased the immunogenicity of Ova, leading to enhanced activation of existing adaptive immunity as well as a greater quantity or quality of newly generated Ova‐specific T cells, which possibly contributed to effective antitumor immunity.

### 2.6 Modification Mapping Unraveled Three Distinct Hyperoxidized Regions in oxOva

**Figure 7:**
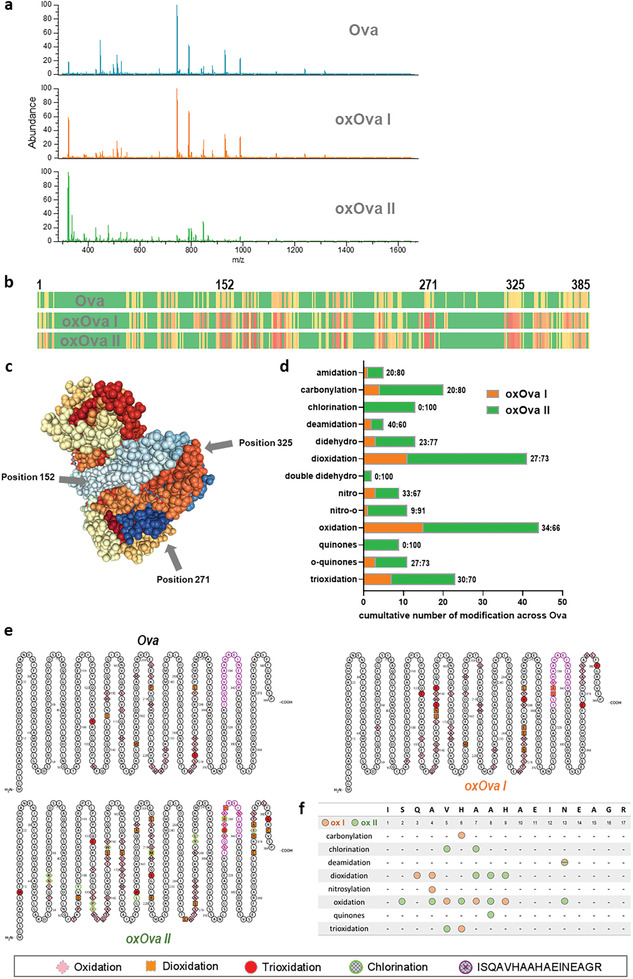
Modification mapping unraveled three distinct hyperoxidized regions in oxOva. a) Mass spectrometry peak distribution of Ova, oxOva I, and oxOva II; b) sum of modifications across the amino acid sequence of Ova, oxOva I, and oxOva II; c) rendering of Ova protein showing the three distinct hyperoxidized positions at outward‐pointed regions of the protein; d) cumulative numbers of different types of post‐translational modifications (PTMs) of oxOva I and oxOva II that were subtracted by the number of background PTMs of native Ova, and relations (%) of oxOva I to oxOva II among the sum of PTMs observed in both oxOva I and II; e) hyperoxidation (oxidation, dioxidation, and trioxidation) in oxOva I and II compared to Ova across its amino acid sequence; f) oxPTMs along the antigenic peptide sequence of Ova. Data are representative of three independent experiments.

The altered immunological perception of oxOva compared to native Ova prompted us to analyze the gas plasma‐introduced post‐translational modifications (PTMs) of the protein using mass spectrometry. The comparison of the chromatograms already revealed major differences in the peak distribution between Ova, oxOva I, and oxOva II (**Figure** **7**a). Subsequent mapping of several types of PTMs to the amino acid sequence of Ova showed several distinct PTM‐hot spot regions (Figure 7b) that appeared at amino acids at the exterior of the protein (Figure 7c). A detailed view of the cumulative number of PTMs in oxOva I and oxOva II was set up next. For each type of PTM, the total number of PTMs in Ova was subtracted from those identified for oxOva I and oxOva II, respectively, and put into relation (percentage of oxOva I or oxOva II of the sum of modifications from oxOva I and oxOva II; Figure 7d). These data were generated based on the assumption that the location and the total number of modifications are possibly relevant to the immunological perception of a protein. oxOva II vaccination showed superior protection from melanoma growth in vivo compared to oxOva I. This correlated with exclusive PTMs for oxOva II (chlorination, quinones, and double didehydro) as well as the majority of total PTMs identified in oxOva I and II together being attributed mostly to oxOva II (>75% for amidation, carbonylation, didehydro, and nitro‐o). Another apparent hallmark of oxOva II was its substantial hyperoxidation (oxidation, dioxidation, and trioxidation) at several regions of the protein sequence (Figure 7e), which was greater in oxOva II as compared to Ova and oxOva I. Subsequently, we performed a more detailed analysis of the 17 amino acid‐long antigenic peptide region of Ova responsible for activating the majority of Ova‐specific CD4^+^ T‐helper cells in OT‐II mice (Figure 7f). Among the modifications mapped out, the amino acids at positions 4 to 9 seemed especially vulnerable to oxidative modifications introduced via the ox I or ox II gas plasma treatment. It was also interesting to note that except for the deamidation at position 13, there was no overlap of modifications in this antigenic sequence between the ox I and ox II condition. This underlined the notion of the different chemistries observed in the ox I versus ox II gas plasma condition (Figure 1) and might relate to the differences observed in the functional consequences of oxOva I and oxOva II in the preventive vaccination experiments (Figure 6). In general, we have investigated only nonenzymatic but not enzymatic PTMs in gas plasma‐treated Ova using mass spectrometry analysis.

## 3 Discussion

The multi‐ROS/RNS environment in chronic inflammation is linked to autoimmunity and cancerogenesis. While the cellular pathobiology in these conditions has been studied extensively during the past decades, the role of ROS/RNS‐derived oxidative modifications received considerably less attention. Using gas plasma, an electron‐impact and photon‐driven technology, we mimicked the multi‐ROS/RNS inflammatory environment and tested the immunological consequences of oxidatively modified ovalbumin (oxOva). This model protein is frequently used for testing the activation of anti‐Ova T cells from genetically engineered mice, and oxOva showed superior T cell activation compared to native Ova. According to the sequential T cell memory model,^[^ ^69^ ^]^ the oxidation might affect the differentiation velocity of T cells, as in the OT‐II model, all cells should be naïve in the beginning. In wild type mice, oxOva vaccination gave enhanced protection from B16F10‐Ova melanoma growth, and mass spectrometry attributed a specific set of oxidative post‐translational modifications to our findings.

Transgenic OT‐II and OT‐I mice are particularly useful for immunological studies of Ova, as these mice harbor Ova‐specific CD4^+^ and CD8^+^ T cells, respectively. Their activation is not dependent on the NOX‐derived production of ROS/RNS, at least with B cells as APCs.^[^ ^70^ ^]^ We noted that oxOva markedly increased CD4^+^ T cell activation in OT‐II splenocytes but not CD8^+^ T cell activation in OT‐I splenocytes. This, in turn, might be due to differences in processing routes being less sensitive to protein oxPTM‐induced alterations, as a link between ROS/RNS, autophagy and immune response had been recently established.^[^ ^71^ ^]^ Exogenously supplied Ova needs to be endocytosed via the mannose‐receptor in dendritic cells (DCs) and additionally the scavenger receptor in macrophages before cross‐presentation via MHC I eliciting CD8^+^ T cell activation. By contrast, pinocytosis is the primary mechanism for MHC II peptide presentation, having a more efficient antigen processing activity, at least in DCs.^[^ ^72^, ^73^ ^]^ Along similar lines, it is also conceivable that the effect of oxPTMs is conserved through lysosomal or endosomal compartments. OT‐II‐derived CD4^+^ T cells recognize Ova peptide at position 323–339 in the context of H‐2A^b^, while OT‐I‐derived CD8^+^ T cells recognize Ova peptide at 257–264 in the context of H‐2K^b^. Gas‐plasma‐mediated oxidative modifications occurred primarily in the amino acid sequence cognate to CD4^+^ but not in that cognate to CD8^+^ T cells, suggesting that oxPTMs might be able to alter the MHC‐peptide‐TCR binding affinity, a crucial factor in T cell activation.^[^ ^74^ ^]^

The binding affinity of amino acids between antigenic peptides and the MHC binding groove is relevant regarding the threshold activation between the TCR and MHC_._^[^ ^75^, ^76^ ^]^ Based on the mentioned observations, an altered binding affinity to MHC loaded with oxidatively modified peptides might be suggested due to oxPTMs in the TCR specific sequence ISQAVHAAHAEINEAGR (Ova_323‐339_) that we determined via mass spectrometry and were associated with an increased T cell activation. The oxidation occurs prototypically at thiols, which respond vastly to gas plasma‐derived ROS and RNS,^[^ ^77^ ^]^ as do other amino acids.^[^ ^78^, ^79^ ^]^ In a previous study, the immunogenicity of an antigenic peptide in its reduced and oxidized state was investigated. The reduced peptide elicited T cell activation, while the oxidized form failed to do so,^[^ ^80^ ^]^ which would contrast our results. In our case, however, there was no cysteine in the antigenic peptide sequence of Ova. In another study, the antigenic peptide for insulin was found to dimerize upon oxidation of cysteine, changing its secondary structure, and directly leading to activation of *γδ* but not *αβ* T cell hybridomas in the case of dimers but not monomers.^[^ ^81^ ^]^ Nevertheless, in our study, we oxidized the entire protein that included the peptide sequence, ruling out the peptide sequence's dimerization upon oxidation as a mechanism of action. However, it is possible that upon Ova cleavage during intracellular antigen processing, dimerization occurred, but it is unclear how this might have contributed to the elevated T cell activation observed. Other reports suggested methionine oxidation for abrogating CD4^+^ T cell activation ^[^ ^82^ ^]^ as well as CD8^+^ T cell activation,^[^ ^83^ ^]^ but our peptide sequence neither contained methionine nor was its oxidation associated with less T cell activity. Interestingly, APCs actively and intracellularly reduce cystines in oxidized antigenic peptides 2–4 h after internalization, leading to enhanced T cell activity,^[^ ^84^ ^]^ as indicated by another report also.^[^ ^85^ ^]^ The importance of cysteine oxidation and its detrimental effects on T cell activation has been recently reported for tumor‐reactive CD8^+^ T cells as well.^[^ ^86^ ^]^ In addition, oxidation was shown to lead to conformational changes in a malaria‐related antigen and reduced T cell activity.^[^ ^87^ ^]^ The studies mentioned above focused on cysteine residues in peptide sequences that were partially oxidized through various (not gas plasma) methods, which might have also oxidized other amino acids in the antigenic peptides, being associated with decreased T cell activation. This suggests that oxidation was less important in explaining our results when considering the 15 oxidations at different amino acids identified in oxOva I or oxOva II. Alternatively, nitrosylation of antigen was reported many times to elicit autoimmunity.^[^ ^88^, ^89^, ^90^, ^91^ ^]^ In our work, gas plasmas produced ^−^ONOO and other RNS might have contributed to the 3‐nitrotyrosine formation. Nevertheless, nitrosylation was only observed in oxOva I but not oxOva II exposure in the antigenic Ova peptide sequences, failing to explain our results fully. This is also the case for chlorination, which was previously shown to enhance antigen immunogenicity,^[^ ^92^ ^]^ but was only found in the ox II treatment that generated HOCl, while ox I did not. However, also deamidation was associated with increased binding affinity of self‐epitopes to HLA,^[^ ^92^ ^]^ and both gas plasma regimens induced this modification at asparagine. This suggests the possibility of oxPTMs in the antigenic peptide sequence to contribute to the enhanced T cell activation observed in our study. In addition, many other oxPTMs were observed across the entire protein that might have led to changes in T cell activation, as previously reported for chlorinated ovalbumin.^[^ ^93^, ^94^ ^]^ At the same time, it is known that peptides with substituted amino acids change the conformational plasticity, leading to altered affinity in MHC‐TCR interaction.^[^ ^95^, ^96^, ^97^ ^]^ Yet, our mass spectrometry analysis did not identify amino acid substitution with gas plasma treatment, ruling out changes in the MHC‐TCR binding via this mechanism.

The presence of APCs was necessary for enhanced T cell activation with oxOva, as magnetically sorted CD4^+^ T cells without APCs failed to be activated in response to Ova or oxOva. Together with a lack of increased activation in DCs and macrophages, this led us to conclude that the gas‐plasma hyperoxidized Ova did not pertain to an inherent DAMP (damage‐associated molecular pattern) activity, at least in our model. This is in contrast to other modifications such as advanced glycosylation end‐products (AGE) on naturally occurring carbohydrates of Ova that are capable of increasing DC activation in a scavenger and mannose receptor‐dependent and RAGE and galectin‐3 independent manner.^[^ ^98^ ^]^ Protein aggregation, observed to a minor extent in our study, also affects protein immunogenicity.^[^ ^99^ ^]^ Aggregation in vaccination development is associated with decreased immunogenicity ^[^ ^100^ ^]^ while protein‐protein‐aggregates (e.g., Ova and TLR agonist) mount markedly elevated DC activation and T cell responses in experimental models.^[^ ^101^ ^]^ Protein aggregation also can lead to autoantibody formation due to the formation of neoepitopes.^[^ ^102^, ^103^ ^]^ For oxidative modifications, previous reports pointed to a pivotal role of Ova chlorination in enhancing its immunogenicity and endocytic uptake as well as intracellular degradation.^[^ ^94^, ^104^ ^]^ However, our oxOva I condition was void of chlorination but still facilitated enhanced CD4^+^ T cell activation in OT‐II mice, pointing to chlorination being one of several factors of elevated gas plasma‐induced protein immunogenicity that involved other types of oxidative modifications.

OxOva II vaccination led to enhanced T cell activation in OT‐II mice and showed superior protection from B16F10‐Ova melanoma growth in wild type mice compared to vaccination with native Ova. This suggested oxOva II to mount a more pronounced antitumor adaptive immune response in terms of either quantity (amplified T cell responses) or quality (broadened anti‐Ova TCR repertoire) or both. Such finding was also reported in a previous study using oxidized whole‐tumor‐lysates fed to DCs used as an autologous vaccine in ovarian cancer patients that strengthened existing T cell responses and led to the generation of T cells targeting cancer neoepitopes.^[^ ^13^ ^]^ Alternatively, it is conceivable that our results emerged from an inflammatory self‐amplification loop initiated by T_H_17 cells that, in turn, aided in the generation of additional antitumor CD8^+^ T cell entities.^[^ ^105^ ^]^ This is supported by our findings that i) oxOva II but not oxOva I vaccination dampened tumor growth in vivo and ii) oxOva II but not oxOva I elicited a T_H_17 cytokine signature both ex vivo and in vivo in OT‐II mice. Noteworthy, also AGE pyrraline‐modified Ova was found to increase IL17A release in Ova‐specific T cells, while – similar to our results – failed to increase DC activation per se.^[^ ^106^ ^]^ IFN*γ* is a molecule known to upregulate MHC I expression in B16F10 melanoma cells,^[^ ^107^ ^]^ and elevated numbers of CD4^+^ TILs were reported to be essential promotors of cancer immunotherapies.^[^ ^108^ ^]^ Our observed increase of IFN*γ* secretion in oxOva conditions hence suggested a stronger immunorecognition of oxOva II compared to native Ova, which possibly altered the tumor microenvironment (TME) in favor of anticancer immunity. In the light of Ova peptide‐MHC complexes being present for at least 16 h on the surface of DCs,^[^ ^109^ ^]^ sustained anti‐Ova T cell generation with oxOva II vaccination might have also contributed to improved antitumor immunity. Vice versa, B16F10‐Ova melanomas are capable of conditioning cancer‐associated fibroblasts to repress CD8^+^ responses within TME T cell zones,^[^ ^110^ ^]^ a mechanism not explored in our study. However, the increased presence of intratumoral DCs in oxOva II vaccinated mice suggested beneficial conditioning of the TME.^[^ ^111^, ^112^ ^]^

Another mechanism that might contribute to the enhanced antitumor effect of oxOva vaccination is intracellular ROS/RNS of tumor cells.^[^ ^113^ ^]^ It is established that cancer cells display elevated ROS/RNS levels due to exacerbated metabolic activity and dysregulated redox balance.^[^ ^114^ ^]^ We hypothesized these ROS/RNS to introduce a more diverse set of modifications in intracellular protein antigens,^[^ ^115^ ^]^ leading to more a diverse set of cognate MHC I peptides than generated with regular unmodified protein vaccines, which elicit a presumably smaller TCR repertoire in the host. Using gas plasma technology, we aimed to bridge this “oxidation gap” by supplying antigen with a maximal diverse set of oxidative modifications capable of mimicking both intracellular and extracellular inflammation‐derived ROS/RNS.^[^ ^116^, ^117^, ^118^, ^119^, ^120^ ^]^ Using cysteine as a model biomolecule, we previously established unique gas plasma‐derived oxidative/nitrosative fingerprints otherwise not yielded with conventional chemical oxidation/nitration methods and the vital role of the plasma feed gas compositions governing and tuning the multi‐ROS/RNS compositions expelled by the plasma jet.^[^ ^77^, ^121^ ^]^ A summary of the many types of ROS/RNS identified in the plasma gas phase and treated liquids was given recently.^[^ ^122^, ^123^ ^]^ Nevertheless, the multi‐ROS/RNS nature of plasma technology poses technical challenges in identifying single agents responsible for the effects observed. This is exemplified in the diverse quality and quantity of oxidative modifications that we identified to be introduced in a protein‐based on a custom‐engineered mass spec oxPTM library generated in‐house.^[^ ^60^ ^]^ However, the identification and exploitation of this angle of protein immunogenicity still is in its infancy. Dramatic differences were observed, e.g., for gas plasma‐treated Ova oxidation/dioxidation/trioxidation in amino acids across the entire protein – types of modifications, a recent autoimmunity ligandomic study, for instance, excluded explicitly in its mass spectrometry peptide analysis workflow.^[^ ^124^ ^]^ We found these types of oxidations to align in the Ova_323‐339_ (CD4^+^) but not the Ova_257‐264_ (CD8^+^) peptide‐binding region, apart from other oxidation hot spots located at three distinct hyperoxidized regions of Ova. A previous report also established that such an oxidation pattern observed in distinct sets of MHC‐peptides not only is a product of random oxidative stress but also serves dedicated redox signaling functions,^[^ ^125^ ^]^ underlining the notion of our oxPTMs signatures related to eliciting distinct immunobiological consequences as observed in vivo. The importance of oxidation and chlorination, as outlined above, has also been pinpointed in a cohort of autoimmune type I diabetic patients that was demonstrated to have autoantibodies targeting experimentally oxidized or chlorinated insulin in vitro.^[^ ^126^ ^]^

A role of RNS‐derived PTMs was implemented as well since we observed nitro and nitro‐o modifications. For instance, nitro fatty acids were identified as bioactive lipids being part of physiological homeostasis as well as metabolic and inflammatory disease,^[^ ^127^ ^]^ exerting signaling functions in cells,^[^ ^128^ ^]^ and promoting the formation of protein PTMs.^[^ ^129^ ^]^ The RNS ^−^ONOO is known to oxidize target molecules and nitrate, e.g., tyrosine and tryptophan efficiently,^[^ ^130^ ^]^ and nitrated protein PTMs have been linked to several inflammatory diseases.^[^ ^131^ ^] –^ONOO, a species suggested to be generated in previous gas plasma‐related studies,^[^ ^36^, ^62^, ^132^, ^133^, ^134^ ^]^ can also form S‐nitrosothiols ^[^ ^135^ ^]^ exhibiting biological functions such as inhibition of NADPH oxidases ^[^ ^136^ ^]^ and release of NO.^[^ ^137^ ^]^ The poor oxidant NO, a species also generated by the gas plasma jet kIPNen,^[^ ^138^ ^]^ can be oxidized to the radical nitrogen dioxide ^[^ ^139^ ^]^ that efficiently nitrates proteins relevant in autoimmunity and cardiovascular disease.^[^ ^140^, ^141^ ^]^ These data emphasize the importance of ROS/RNS‐derived PTMs in health and disease and suggest more findings to come if the multi‐ROS/RNS nature of inflammation would be recapitulated using gas plasma systems. However, and owing to both a large number of targets on proteins and the complexity of antigen uptake and presentation, a direct and causative link between an individual or a set of oxPTMs and immunorecognition remains to be established.

## 4 Conclusion

Gas plasma, an electron‐impact and photon‐driven technology, was employed to generate a diverse range of ROS/RNS simultaneously for testing their effect on protein oxidative post‐translational modifications (oxPTMs) and immunogenicity. Using ovalbumin (Ova) as a model protein, we confirmed increased immunogenicity of oxOva by detecting higher amounts of activated T cells correlating with decreased tumor burden and a broad set of nonenzymatic Ova‐oxPTMs with oxidation and chlorination suggested to be of prime importance. Our proof‐of‐concept study has expansive implications for further research in autoimmunity and vaccine research.

## 5 Experimental Section

##### Plasma Treatment

Lyophilized EndoGrade ovalbumin (Ova; Hyglos, Germany) was solved in double‐distilled water (ddH_2_O) and diluted in PBS (100 µg mL^−1^). In the setup, the protein does not exert a specific function, such as enzymatic activity. Instead, it serves as immunostimulant once taken up by antigen‐presenting cells and presented to antigen‐specific T cells. In some experiments, *n*‐acetylcysteine (NAC, 2 × 10^−3^ m, Thermo Fisher, USA) was added before gas plasma treatment of 500 µl suspensions in a 24‐well plate (Sarstedt, Germany). In additional control experiments, the Ova suspension was exposed to four pulses of electric fields (ECM 830, BTX, USA) at 1.5 kV cm^−2^. For gas plasma treatment, the kINPen plasma jet (neoplas, Germany) was used, which is accredited as a biomedical device in Europe. The atmospheric pressure gas plasma jet requires a DC power unit. A ceramic capillary with an inner diameter of 1.6 mm has mounted at its center a pin‐type electrode with a diameter of 1.0 mm diameter. A radiofrequency generator produces a sinusoidal voltage waveform, ranging from 2 to 3 kV amplitude peak at a frequency of 1 MHz. The gas plasma is generated at the tip of the central electrode and expands into the ambient air. The UV irradiation of the gas plasma jet is 105 µJ cm^−2^. A more detailed description of the chemistry and physics of the device was recently provided.^[^ ^122^ ^]^ In our study, the gas plasma jet was operated at two standard liters per minute of either pure argon gas (3 min treatment time) or helium gas containing 2% oxygen (1 min treatment time). Gases were 99.999% pure and from Air liquid, France. For investigating plasma‐derived products, the plasma jet was positioned at a distance of 0.8 cm and perpendicular to the front of a UV‐sensitive optical emission spectrometer (AvaSpec‐2048‐USB2; Avantes, Germany) with a spectral resolution of 0.7 nm. The OES lens center was aligned to the visible tip of the plasma, which was 0.8 cm for the argon condition and 0.6 cm for the helium/oxygen condition. A computer‐controlled *xyz* motorized table ensured the plasma jet's exact positioning in this setup (CNC step, Germany). This setup was also used to attain sub‐millimeter precision to maximize the reproducibility of the gas plasma treatment of samples residing in multiwell plates. Gas flux‐mediated evaporation of treated liquids was compensated for by adding a predetermined amount of ddH_2_O.

##### ROS/RNS and Liquid Analysis

During plasma treatment, the temperature of the liquid was analyzed using a PIX infrared camera (Optrix, Germany). PBS was supplemented either with or without Ova or *n*‐acetylcysteine (NAC, 2 × 10^−3^ m; Thermo Fisher, USA) before plasma treatment. Singlet oxygen was measured using singlet oxygen sensor green (Thermo Fisher, USA), and its fluorescence was analyzed at *λ*_ex_ 485 nm and *λ*_em_ 535 nm using a plate reader as described before.^[^ ^142^ ^]^ Nitrite and nitrate were determined by performing the Griess‐assay (Cayman Chemical, Germany) as previously outlined,^[^ ^41^ ^]^ and absorbance was measured at *λ*_ex_ 548 nm. Hydrogen peroxide was quantified using the Amplex Ultra Red detection reagent (Thermo Fisher, USA) according to previous protocols,^[^ ^143^ ^]^ and measured at *λ*_ex_ 535 nm and *λ*_em_ 590 nm using a plate reader. Hypochlorous acid generated via the gas plasma was measured using the taurine chloramine assay at an absorption of 645 nm, as outlined before.^[^ ^35^ ^]^ Aminophenyl fluorescein (APF) and hydroxyphenyl fluorescein (HPF; both Thermo Fisher, USA) sense HOCl, peroxynitrite (^−^ONOO), and hydroxyl radicals (^.^OH), and ^−^ONOO and ^.^OH, respectively.^[^ ^61^ ^]^ The dyes were used at a final concentration of 5 × 10^−6^ m, and their fluorescence was analyzed at *λ*_ex_ 485 nm and *λ*_em_ 535 nm. Also, diaminofluorescein (DAF, final concentration 5 × 10^−6^ m; Thermo Fisher) was analyzed this way. Hydroxyl radicals were measured using the terephtalic acid assay as described before.^[^ ^146^ ^]^

##### Circular Dichroism (CD) Spectroscopy

Native and plasma‐treated ovalbumin in PBS (100 µg mL^−1^) with or without NAC (2 × 10^−3^ m) was measured via CD‐spectroscopy using a Chirascan V100 CD Spectrometer (Applied Photophysics, UK). Samples were loaded in 5 mm‐pathlength cuvettes (Hellma Analytics, Germany). Spectra were recorded at 20 °C over a wavelength range from 190 nm to 270 nm with a bandwidth of 1.0 nm and a scanning time of 1.5 s per point. Measurements were repeated five times. All spectra are blank corrected.

##### Photon Correlation Spectroscopy

Photon correlation spectroscopy measurements of native or gas plasma‐treated Ova (100 µg mL^−1^) using a ZS90 dynamic light scattering (DLS) device (Malvern instruments, USA) equipped with a helium‐neon laser light source (632 nm). Proteins (Material RI = 1.45, absorption = 0.001) in PBS similar to water (Dispersant RI = 1.33, viscosity = 0.954) were measured in low‐volume disposable cuvettes (ZEN0040). DLS measurements were done at a set angle of 90° and attenuator at 11. The size was measured at 22 °C, with an equilibration time of 120 sec and cuvette position at 3 mm. Backscatter angled detection was performed at 173° with a scattering collection angle of 147.7°. Each biological replicate was measured in several replicates with minimal time between repeats. Data analysis was carried out from three independent experiments.

##### B16F10‐Ova

Ova‐expressing murine melanoma cells (B16F10‐Ova) were a kind gift of Karl Sebastian Lang (Institute of Immunology, University Hospital Essen, Germany). Cells were cultured in Roswell Park Memorial Institute (RPMI) 1640 medium (PanBioTech, Germany) containing 10% fetal bovine serum, 2% glutamine, 1% penicillin/streptomycin (all Sigma, Germany), and 0.5 µg mL^−1^ puromycin (StemCell Technologies, Germany). Cells were grown at 37 °C, 95% humidity, and 5% CO_2_, and subcultured three times a week.

##### In Vivo Experiments and Cell Isolation

Ethical approval was received from the local authority (*Landesamt für Landwirtschaft, Lebensmittelsicherheit und Fischerei* in the state of Mecklenburg‐Vorpommern, Germany; approval number M‐V 7221.3‐1‐022/19). C57BL/6N, C57BL/6‐Tg(TcraTcrb)1100Mjb/Crl (OT‐I), and C57BL/6‐Tg(TcraTcrb)425Cbn/Crl (OT‐II), all female at 6–8 weeks of age, were purchased (Charles River Laboratories, Germany). Transgenic OT‐II mice harbor T cells with specific receptors for the Ova peptide ISQAVHAAHAEINEAGR (Ova_323‐339_) restricted by MHC‐II (I‐Ab),^[^ ^144^ ^]^ and transgenic OT‐I mice harbor CD8^+^ T cells specific for the Ova peptide SIINFEKL (Ova_257‐264_) restricted by MHC‐I (H_2_D_b_).^[^ ^145^ ^]^ Animals were kept in cages with a maximum of six animals per cage. OT‐II and C57BL/6N mice received intraperitoneal injections of 100 µl of PBS with or without 10 µg of Ova or gas plasma‐treated Ova (oxOva I for Ar plasma; oxOva II for He/O_2_ plasma). C57BL/6N received a boost vaccination (without adjuvant) seven days later. On day 10, animals were challenged with a subcutaneous inoculation of 1 × 10^4^ B16F10‐Ova melanoma cells. After sacrifice, lymphoid organs and tumors of tumor‐bearing C57BL/6 animals were removed. Splenocyte isolation and tumor digestion were performed using the splenocytes isolation kit and tumor dissociation kit, respectively, in an OctaMACS Dissociator device (Miltenyi Biotec, Germany). CD4^+^ were separated from untouched splenocytes via a negative selection kit containing antibodies against CD8a, CD11b, CD11c, CD19, CD24, CD45R/B220, CD49b, CD105, I‐A/I‐E (MHC II), TER‐119/Erythroid, and TCR‐*γδ* (BioLegend, UK). MojoSort cell separation was performed according to the manufacturer's instructions.

##### Restimulation Assay

Isolated splenocytes were resuspended in fully supplemented culture medium. For experiments, 1.5 × 10^6 ^cells in 500 µl of medium were incubated for 24 h with PBS or PBS containing Ova, oxOva I, or oxOva II. In control experiments, only PBS was plasma‐treated and added to splenocytes, followed by the addition of vehicle (PBS) or Ova immediately afterwards. For MHC‐II blocking experiments, isolated splenocytes were preincubated for 1 h with 1 µg mL^−1^ of purified I‐A/I‐E monoclonal antibodies (clone M5/114.15.2; BioLegend, UK).

##### Ovalbumin Uptake

Splenocytes were labeled with fluorescently conjugated antibodies targeted against F4/80 (phycoerythrin, PE; clone BM8; BioLegend, UK) and CD11b (alexa fluor 700; clone M170; BioLegend, UK). DQ‐Ova (5 µg mL^−1^; Thermo Scientific, Germany) was added. The dye exhibits a green fluorescence, which is quenched by aggregation. Upon uptake, aggregation is reduced, enhancing fluorescence emission, which was monitored using fluorescence microscopy (Operetta CLS; Perkin Elmer, Germany). Measurement was performed with a 20x (NA 0.4) objective (Zeiss, Germany) in the brightfield (BF), digital phase contrast (DPC), and fluorescence (*λ*_ex_ 475 nm, 550 nm, and 630 nm) channel. Flow cytometry was performed to analyze DQ‐Ova or DQ‐BSA (both 20 µg mL^−1^; Thermo Scientific, Germany) in previously separated APCs of splenocytes or differentiated monocytes isolated from PBMCs. This study was approved by the local ethics committee (approval number: BB166/17). Whole blood was drawn from volunteers with informed consent. Human cells were labeled with fluorescently conjugated antibodies targeting CD11c (Brilliant Violet 510, clone 3.9), HLA‐DR (APC‐Cy7) and ZOMBIE NIR for dead cell exclusion (all BioLegend, Netherlands), and incubated with DQ alone or in combination with native or plasma‐treated proteins (10 µg mL^−1^).

##### Multiplex Cytokine Analysis

Cytokines were measured in supernatants of minced lymph nodes from in vivo experiments with OT‐II mice and supernatants of OT‐II‐derived splenocytes cultured ex vivo with Ova or oxOva for 24 h, using multiplex cytokine detection technology (LegendPlex; BioLegend, UK) according to the manufacturer's instructions. This plex is a bead‐based sandwich immunoassay and was measured using flow cytometry (CytoFLEX S; Beckman‐Coulter, USA) targeting interferon‐gamma (IFN*γ*), tumor necrosis factor‐alpha (TNF*α*), and eleven interleukins (IL‐2, IL‐4, IL‐5, IL‐6, IL‐13, IL17A, IL‐17F, IL‐21, IL‐22, IL‐9, and IL‐10). For quantification, data analysis software (Vigene Tech, France) was utilized. A separate standard curve was calculated using fifth‐degree polynomials for each analyte, with attention to the analytes’ specific detection limits.

##### Flow Cytometry

Cells were collected in FACS tubes and washed three times with cold FACS washing buffer (Miltenyi Biotec, Germany). For live/dead discrimination and T cell analysis, cells were stained with activated Caspase 3/7 detection reagent (Thermo Scientific, USA) and fc block (BioLegend, UK) at room temperature for 10 min followed by incubation with fluorescently conjugated monoclonal antibodies targeting CD62L (PE‐Dazzle, clone MEL‐14), CD44 (PerCP‐Cy5.5, clone IM7), CD4 (PE‐Cy7, clone L3T4), CD25 (APC, clone PC61), CD3 (Alexa Fluor 700, clone 17A2), CD69 (brilliant violet 421, clone H1.2F3), and CD8 (brilliant violet 510, clone 53–6.7) (all BioLegend, UK) for 30 min at 4 °C. For T cell analysis, unwanted cells were gated out using a dump channel containing zombie‐NIR as well as CD45R and I‐A/I‐E APC‐fire 750 (BioLegend, UK). For macrophage analysis, leftover suspension cells were transferred into a tube, and attached cells were scratched‐off and added to FACS tubes. All cells were washed as described above and stained for 30 min at 4 °C with fluorescently conjugated monoclonal antibodies targeting F4/80 (PE, cone BM8), CD11b (PE‐Dazzle, clone M1/70), CD86 (PE‐Cy7, clone PO3), CD64 (APC, clone X54‐5/7.1), I‐A/I‐E (AF700, clone M5/114.15.2), CD45.2 (APC‐Cy7, clone 104), Ly6G (APC‐Cy7, clone HK1.4), CD24 (BV421, clone M1/69), and CD11c (BV605, N418). Dead cells were excluded using Sytox green dye (Thermo Scientific, Germany). After washing with cold FACS washing buffer, samples were measured using flow cytometry (CytoFLEX S and CytoFLEX LX; Beckman‐Coulter, USA). Data analysis was performed using Kaluza analysis software 2.1 (Beckman‐Coulter, USA).

##### Proliferation Assay

For cell proliferation experiments, cells were labeled with carboxyfluorescein succinimidyl ester (CFSE, 2.5 × 10^−6^ m; ThermoFisher, Germany), and exposed ex vivo to either Ova, oxOva I, or oxOva II, or to native human albumin, argon gas plasma‐oxidized human albumin (oxhuAlb I), or helium/oxygen gas plasma‐oxidized human albumin (oxhuAlb II) as controls. Three days later, cells were collected, washed, and labeled with antibodies to identify proliferating CD4^+^ T cells as well as their activation and differentiation status (CD69, CD25, CD44) among all TCRvB^+^ cells. Sample acquisition was performed using an LSR II flow cytometer (Becton‐Dickinson, USA) and analyzed using Flow Jo (TreeStar Software, USA).

##### Gel Electrophoresis and Western Blot

All reagents, buffers, and devices were supplied by ThermoFisher Scientific unless otherwise stated. 30µl of PBS containing native or gas plasma‐treated Ova (30 µg for coomassie, 15 µg for western blot) were mixed with 4x NuPAGE LDS sample buffer and loaded without denaturation on a 10‐well 4–12% Bis‐Tris Gel. SeeBlue prestained standard was loaded, and gel electrophoresis was performed in a chamber filled with 1x MES SDS running buffer and connected to a power supply (Biometra Analytik‐Jena, Germany). For coomassie, gels were stained with 4% coomassie brilliant blue R250 in 80% methanol, 20% acetic acid (both Carl Roth, Germany), and washed with a de‐staining solution (20% methanol, 10% acetic acid, 70% ddH_2_O). For western blot, proteins were blotted on an activated PDVF membrane, blocked with Rotifix (Carl Roth, Germany), and stained with anti‐Ova polyclonal primary antibody (Biozol, Germany) followed by secondary horse‐radish peroxidase‐coupled antibodies (Rockland Immunochemicals). Signals were acquired after adding ECL reagent super signal WestPicoPlus in a chemiluminescence detection system (GE Healthcare, USA).

##### Mass Spectrometry and Data Analysis

Gas plasma‐mediated protein modifications were investigated using high‐resolution mass spectrometry coupled to liquid chromatography (LC/MS). Samples were prepared for LC/MS analysis by adding four volumes of acetone (Sigma, all chemicals of MS grade). After overnight incubation at −20 °C, samples were centrifuged at 12.000 x *g* for 20 min, the supernatants were removed, and the dried pellet was solved in ddH_2_O. After the determination of protein concentration using the Bradford assay (RCDC assay, BioRad), 100 µg of protein was reduced in 50 × 10^−3^ m TEAB buffer (Sigma, Germany) by adding Tris(2‐carboxyethyl)phosphine (Merck, Germany) at a final concentration of 5.7 × 10^−3^ m. After incubation at 60 °C for 45 min, 0.5 × 10^−3^ m iodoacetamide (Merck, Germany) was added, and samples were incubated for 20 min at 22 °C. Proteins were digested by trypsinization for 18 h at room temperature and loaded on STAGE‐tips (Thermo Fisher, USA) filled with 30 µg of Luna C18‐Material (Phenomex, Germany), which were washed and equilibrated beforehand with acetonitrile and water, respectively (both ChemSolv, USA). Desalting was performed by washing twice with 0.1% acetic acid (Merck, Germany) and centrifugation (8000 x g, 1 min). To elute peptides from the STAGE‐tip, 30 µl acetonitrile containing 0.1% acetic acid were added and pressed through the tip using pressurized nitrogen. Acetonitrile was removed by adding 20 µl of 0.1% acetic acid and vacuum centrifugation to a final volume of 10 µl. LC/MS measurements were performed using a Q‐Exactive Orbitrap coupled to an UltiMate 3000 nano HPLC (both Thermo Scientific, USA). Samples were concentrated on a PepMap C18 precolumn (20 mm x 100 µm inner diameter, 5 µm particle size) before peptides were separated on a PepMap C18 column (150 mm x 75 µm inner diameter, 3 µm particle size) running water (eluent A) against acetonitrile (eluent B). Both eluents had 0.1% acetic acid added as a modifier. The gradient was as follows: initial conditions 250 nL min^−1^ flow of 2% B, in 4 min to 10% B, in 20 min to 35% B, in 1 min to 50% B, in 2 min to 80% B. Following the gradient, washing of the column at 80% B for 8 min, followed by equilibration at 2% B for 8 min were performed. Flow for washing and equilibration was ramped to 500 nL min^−1^. The Q‐Exactive was fitted with a Nanospray flex source (Thermo Scientific, USA) and was running in data‐dependent acquisition mode (Top25) with tune parameters adjusted for optimal signal intensities. Data analysis was performed in Proteom Discoverer 2.3 (Thermo Scientific, USA). The initial quality of measured spectra was assessed by searching against a chimeric database containing the ovalbumin sequence as well as the full human proteome as a control for possible contaminants using SequestHT and MS Amanda 2.0 search engines. An in‐depth analysis of modifications was performed using Byonic software (Proteinmetrics, USA) as a plug‐in into Proteom Discoverer running against an in‐house designed database for oxidative and post‐translational modifications.

##### Statistical Analysis

Data are from several independent experiments and show mean and standard error if not indicated otherwise. Statistical analysis was performed using t‐test, one‐way anova, or two‐way anova, as indicated. Asterisks indicate the level of significance as follows: *, **, or *** for the *p*‐values <0.05, <0.01, or <0.001, respectively. Statistical analysis was carried out using prism 8.4 (GraphPad Software, USA).

## Conflict of Interest

The authors declare no conflict of interest.

## Supporting information



Supporting Information

## Data Availability

Research data are not shared.
