# Supplementary material for: Gas Plasma Technology Augments Ovalbumin Immunogenicity and OT‐II T Cell Activation Conferring Tumor Protection in Mice
Source: Adv Sci (Weinh). 2021 Mar 8;8(10):2003395. doi: 10.1002/advs.202003395 (PMC8132054; doi:10.1002/advs.202003395)
Supplement: Supplementary file 1 — Supporting Information [file ADVS-8-2003395-s001.pdf]

© 2021 Wiley-VCH GmbH

ADVANCED SCIENCE
Open Access

Supporting Information

for *Adv. Sci.,* DOI: 10.1002/advs.202003395

Gas plasma technology augments ovalbumin immunogenicity and OT-II T cell activation conferring tumor protection in mice

*Ramona Clemen, Eric Freund, Daniel Mrochen, Lea Miebach, Anke Schmidt, Bernhard H. Rauch, Jan-Wilm Lackmann, Ulrike Martens, Kristian Wende, Michael Lalk, Mihaela Delcea, Barbara M. Bröker, Sander Bekeschus**

Supplemental figure S1. (a) PBS with or without OVA or NAC was exposed as in figure 1, and absolute concentration of hydroxyl radicals were measured as recently as recently described [146]; (**b-c**) NAC was added to Ova before exposure to ox I (oxOva I + NAC) and ox II (oxOva II + NAC) that abrogated oxidation-induced changes in dynamic light scattering (**b**) and protein structure in CD spectroscopy (**c**); (**d**) OT-II splenocytes were incubated with Ova, oxOva I, oxOva II, or Ova exposed to pulsed electric fields, and $CD4^+$ T cells were analyzed for activation 24h later, underlining the inability of pulsed fields alone as a physical trait also observed in plasma jets to promote the immunogenicity of Ova; (**e**) OT-II mice were vaccinated with Ova, oxOva I, or oxOva II that were gas plasma-treated in the presence or absence of NAC, showing reduced T cell activation of re-stimulated $CD4^+$ splenocytes *ex vivo*; (**f-g**) bone marrow-derived dendritic cells from C57BL/6 wildtype mice (n=3) were incubated with Ova or oxOva II, and analyzed for protein phosphorylation by western blot (**f**) yielding no statistically-significant changes for oxOva II when analyzed at 15min, 30min, and 60min post exposure (**g**). Statistical analysis was performed using t-test (* p<0.05; ** p<0.01; *** p<0.001); ns = not significant.

Supplemental figure S1

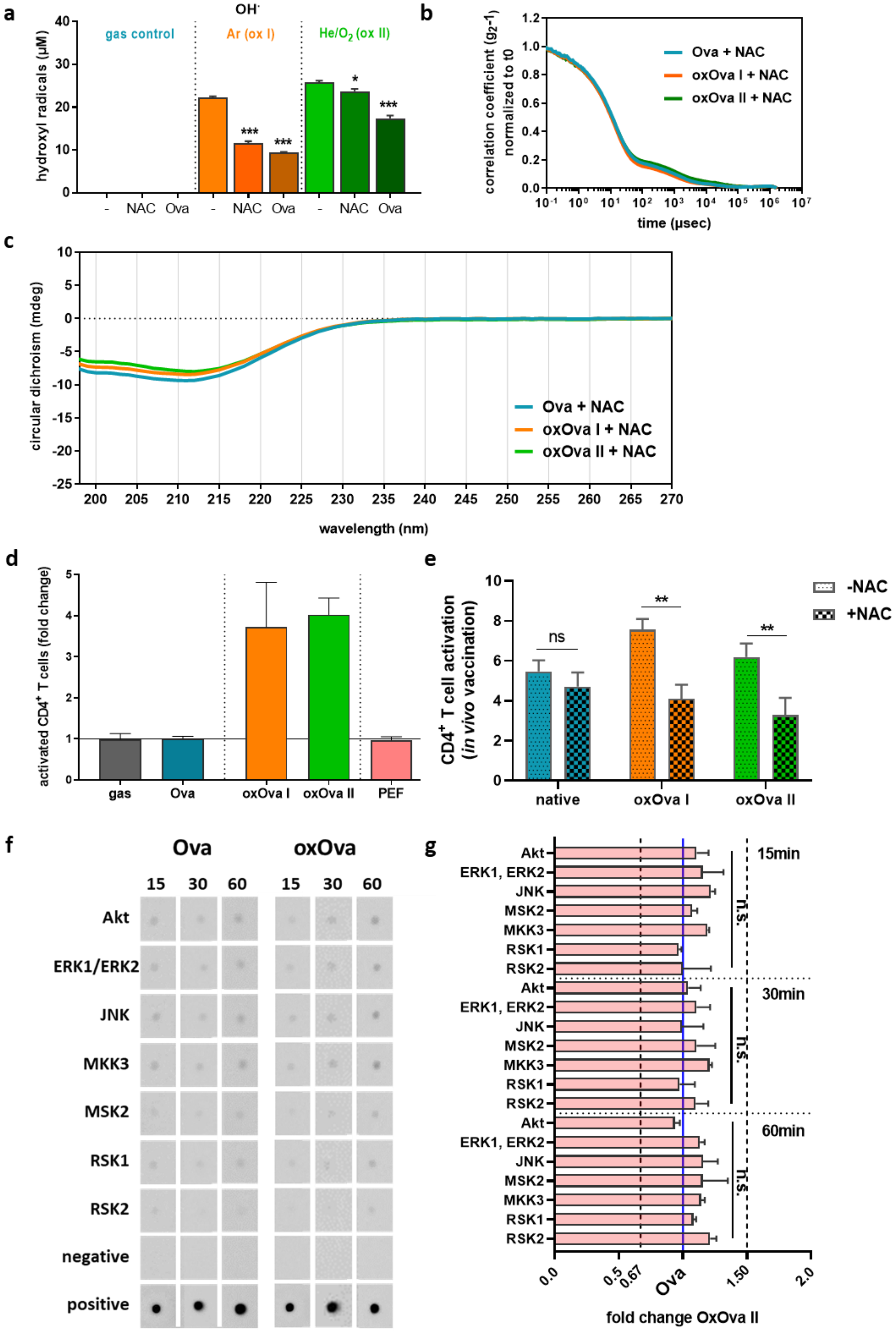
a
OH·
gas control
Ar (ox I)
He/O2 (ox II)
hydroxyl radicals (µM)
- NAC Ova - NAC Ova - NAC Ova
b
correlation coefficient (g2-1) normalized to t0
time (µsec)
Ova + NAC
oxOva I + NAC
oxOva II + NAC
c
circular dichroism (mdeg)
wavelength (nm)
Ova + NAC
oxOva I + NAC
oxOva II + NAC
d
activated CD4+ T cells (fold change)
gas
Ova
oxOva I
oxOva II
PEF
e
CD4+ T cell activation (in vivo vaccination)
-NAC
+NAC
ns
native
oxOva I
oxOva II
f
Ova
oxOva
15 30 60 15 30 60
Akt
ERK1/ERK2
JNK
MKK3
MSK2
RSK1
RSK2
negative
positive
g
Akt
ERK1, ERK2
JNK
MSK2
MKK3
RSK1
RSK2
15min
30min
60min
n.s.
Ova
fold change OxOva II
